# Towards integrated surveillance of zoonoses: spatiotemporal joint modeling of rodent population data and human tularemia cases in Finland

**DOI:** 10.1186/s12874-018-0532-8

**Published:** 2018-07-05

**Authors:** C. Rotejanaprasert, A. Lawson, H. Rossow, J. Sane, O. Huitu, H. Henttonen, V. J. Del Rio Vilas

**Affiliations:** 1 Department of Tropical Hygiene, Faculty of Tropical Medicine, Mahidol University, Ratchathewi, Bangkok, 10400 Thailand; 2 Department of Public Health Sciences, Medical University of South Carolina, Charleston, SC 29425 USA; 3 Department of Veterinary Biosciences, Faculty of Veterinary Medicine, University of Helsinki, Helsinki, Finland; 4 National Institute for Health and Welfare, Helsinki, Finland; 5 Natural Resources Institute Finland, Helsinki, Finland; 6 School of Veterinary Medicine, University of Surrey, Guildford, UK

**Keywords:** Surveillance integration, Joint diseases modeling, Zoonoses, Tularemia, Finland

## Abstract

**Background:**

There are an increasing number of geo-coded information streams available which could improve public health surveillance accuracy and efficiency when properly integrated. Specifically, for zoonotic diseases, knowledge of spatial and temporal patterns of animal host distribution can be used to raise awareness of human risk and enhance early prediction accuracy of human incidence.

**Methods:**

To this end, we develop a spatiotemporal joint modeling framework to integrate human case data and animal host data to offer a modeling alternative for combining multiple surveillance data streams in a novel way. A case study is provided of spatiotemporal modeling of human tularemia incidence and rodent population data from Finnish health care districts during years 1995–2012.

**Results:**

Spatial and temporal information of rodent abundance was shown to be useful in predicting human cases and in improving tularemia risk estimates in 40 and 75% of health care districts, respectively. The human relative risk estimates’ standard deviation with rodent’s information incorporated are smaller than those from the model that has only human incidence.

**Conclusions:**

These results support the integration of rodent population variables to reduce the uncertainty of tularemia risk estimates. However, more information on several covariates such as environmental, behavioral, and socio-economic factors can be investigated further to deeper understand the zoonotic relationship.

## Background

Disease risk mapping is important for the understanding of the spatial epidemiology of infectious diseases. In most cases and even for multi-host diseases such as zoonoses, risk estimation has been conducted in a univariate fashion based on human case data alone. Modeling of multivariate health data, informed by multiple streams of geo-coded information, allows observation of concurrent patterns among data streams and conditioning on one another. As a result, multivariate methods can deliver greater statistical power and lead to more precise risk estimation and enhanced event detection. Specifically, for zoonotic diseases, knowledge of spatial and temporal patterns of the animal host could inform incidence in humans.

Integration of data and analyses, whether of population or health related variables, has been suggested to improve zoonoses surveillance accuracy and efficiency [1, 2]. Integration appears more feasible for endemic zoonoses, and for those with domesticated animals as source, given the likely greater availability of animal health data. For zoonoses with a non-domesticated animal source (e.g. sylvatic yellow fever, tularemia), availability of animal health data is likely to be a limiting factor towards integration and alternative animal data sources must be sought.

Tularemia is an infectious disease caused by an intracellular bacterium, *Francisella tularensis*. The disease is endemic in North America and parts of Europe, with recurrent outbreaks in Sweden and Finland [3, 4]. *Francisella tularensis* has a wide range of hosts with transmission most commonly via arthropod vectors [5]. Rodents could play a role in the zoonotic transmission of the disease after findings of a relationship between vole population cycles and human tularemia incidence in Finland [6] and Sweden [7]. Specifically in Finland, rodent population dynamics displayed a spatiotemporal relationship with human tularemia cases, such that human case numbers peaked one year after peak rodent densities [6]. Similar findings, from studies of tularemia outbreaks, indicate that high rodent densities might relate to occurrences in humans [8–11].

This work explores the application of spatiotemporal joint models to concurrent animal and human geo-referenced data sources in an effort to explain possible patterns between the distribution and/or the abundance of the animal host and human disease.. The proposed methodology is evaluated on its performance in predicting human disease risk and improving risk estimation in a case study of tularemia human incidence and rodent population data in Finland. Not only our method contains methodological novelty with potential applications in spatial epidemiology, it also helps to reveal a disease pattern in the case study which was not considered in previous studies.

## Methods

### Data sources

**Fig. 1:**
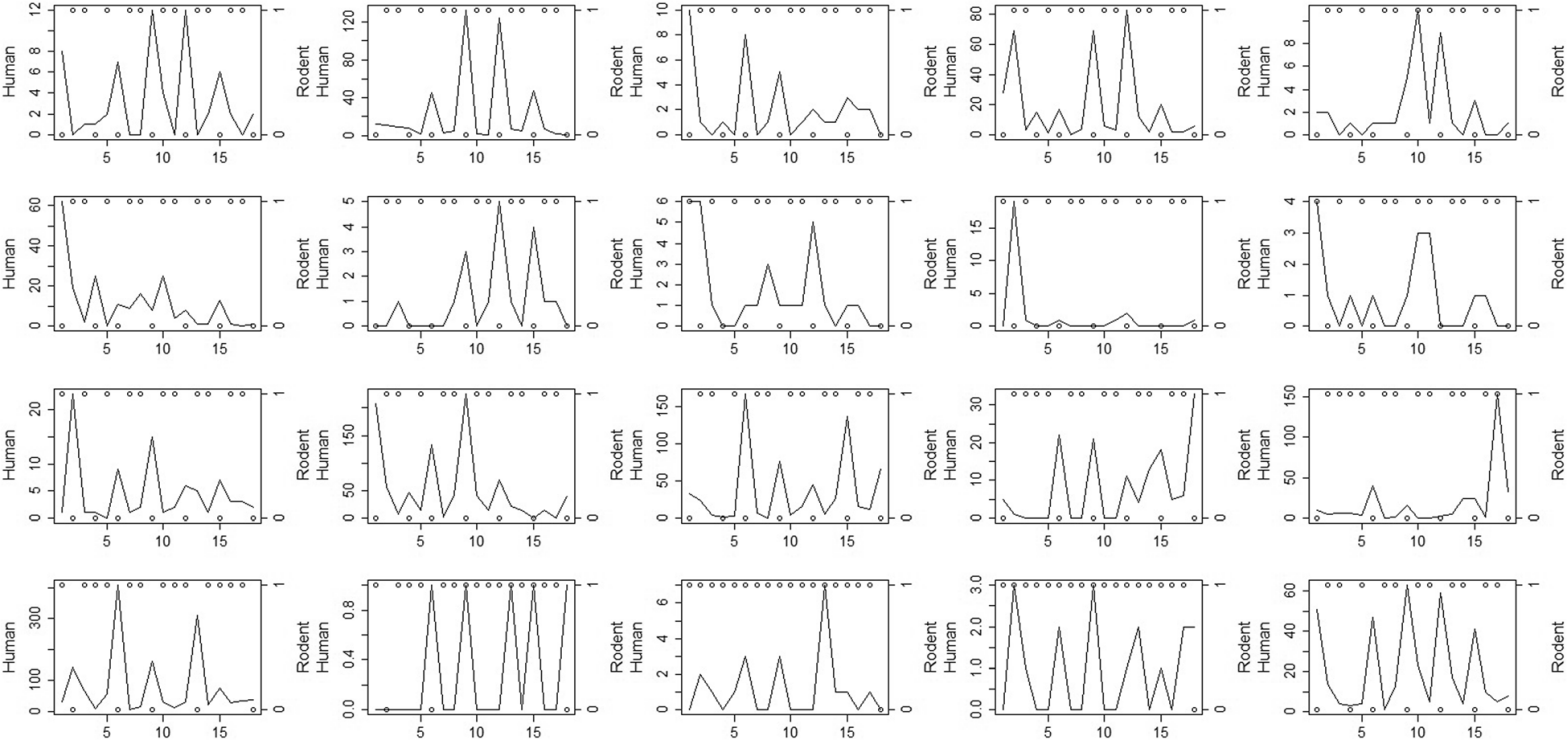
Human cases (solid line) and 2-level rodent status (dot) for the 20 Finnish health districts over years 1995–2012

**Fig. 2:**
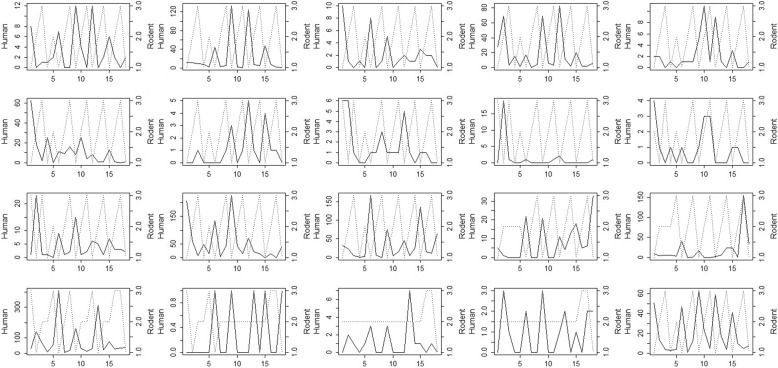
Human cases (solid line) and 3-level rodent status (dotted line) for the 20 Finnish health districts over years 1995–2012

A complete description of the rodent population and human tularemia incidence data is available from earlier reports [6]. Briefly, data on rodent population levels, predominantly bank voles (*Myodes glareolus*) and field voles (*Microtus agrestis*), were collected across Finland by the Natural Resources Institute Finland and categorized into three population levels: decline, increase, and peak [12]. Human tularemia cases were reported as laboratory-confirmed to the National Infectious Disease Register, kept by the National Institute for Health and Welfare of Finland. Both human cases and rodent data were aggregated into 20 Finnish healthcare districts over the period 1995–2012 [6]. An indicator to quantify the influence of rodent population levels on human incidence is developed for each health district. Plots of human cases and binary rodent status for the 20 Finnish health districts in the period 1995–2012 show a one-year lagged increase in the number of human tularemia cases after rodent population peaks for certain districts and years (Fig. 1). A similar pattern was found between human cases and the categorical rodent population status (Fig. 2). These support the choice of spatiotemporal models which will be developed in the next section.

### Statistical methodology

We propose a Bayesian framework to jointly analyze rodent population status and human case incidence. We assume that the human cases are associated with rodents’ status through a latent structure. Let human cases (counts), *h*_*it*_, at health district *i* and time *t* follow a Poisson distribution with mean = *e*_*i*_*θ*_*it*_ where *e*_*i*_ is the expected number of human cases in the *i*th health district (presumably constant across the years) and *θ*_*it*_ is the relative risk at the *i*th health district and year *t* There are a number of ways to calculate the expected rate. In this paper, the expected rates, *e*_*i*_, are calculated as the average case count at area *i* over the time period as ${e}_i=\frac{\sum_t{h}_{it}}{T}$ where *T* is the length of study period (*T* = 8 years)*.* Human population data was obtained from the Finnish Population Register Centre [13]. However, the population variation between regions was found to be limited and combined with the low overall rate of the disease, it was decided that a time averaged rate would be appropriate in this case.

A simple approach to jointly model human incidence and rodent population data is to consider a 2-level rodent population indicator as in [6]. As a binary variable, we denote *r*_it_ = 0 if the number of rodents declined and *r*_it_ = 1 if the number of rodents is at peak or increased. The 2-class rodent status is then assumed to follow a Bernoulli distribution with parameter *p*_*it*_ being the probability of rodent for the *i* th health district and year *t*. To specify the parameters in the joint likelihoods, *θ*_*it*_for humans and *p*_*it*_ for rodents, linear predictors are decomposed additively into spatial, and space-time interaction random effects as follows

$$ {\displaystyle \begin{array}{l}{\mathrm{h}\mathrm{uman}}_{it}={\mathrm{h}}_{it}\sim Poisson\left({e}_i{\theta}_{it}\right)\\ {}\log \left({\theta}_{it}\right)={\alpha}^h+{u}_i^h+{v}_i^h+{\lambda}_t^h+{\delta}_{it}^h\\ {}{\delta}_{it}^h={\beta}_i{\delta}_{it-1}^r\\ {}{\beta}_i={\beta}_i^p{r}_{it-1}+{\beta}_i^n\left(1-{r}_{it-1}\right)\\ {}{\mathrm{r}\mathrm{odent}}_{it}={\mathrm{r}}_{it}\sim Bernoulli\left({p}_{it}\right)\\ {}\log \mathrm{it}\left({p}_{it}\right)={\alpha}^r+{u}_i^r+{v}_i^r+{u}_k^{sr}+{v}_k^{sr}+{\lambda}_t^r+{\delta}_{it}^r\end{array}}\kern0.5em {\displaystyle \begin{array}{l}{\alpha}^h\sim N\left(0,{\tau}_{\alpha^h}^{-1}\right);{\alpha}^r\sim N\left(0,{\tau}_{\alpha^r}^{-1}\right)\\ {}{\delta}_{it}^h\sim N\left(0,{\tau}_{\delta^h}^{-1}\right);{\delta}_{it}^r\sim N\left(0,{\tau}_{\delta^r}^{-1}\right)\\ {}{\lambda}_t^h\sim N\left({\lambda}_{t-1}^h,{\tau}_{\lambda^h}^{-1}\right);{\lambda}_t^r\sim N\left({\lambda}_{t-1}^r,{\tau}_{\lambda^r}^{-1}\right)\\ {}{\lambda}_1^h\sim N\left(0,{\tau}_{\lambda^h}^{-1}\right);{\lambda}_1^r\sim N\left(0,{\tau}_{\lambda^r}^{-1}\right)\\ {}{u}_i^h\sim ICAR\left({\tau}_{u^h}^{-1}\right);{v}_i^h\sim N\left(0,{\tau}_{v^h}^{-1}\right)\\ {}{u}_i^r\sim ICAR\left({\tau}_{u^r}^{-1}\right);{v}_i^r\sim N\left(0,{\tau}_{v^r}^{-1}\right)\\ {}{u}_k^{sr}\sim ICAR\left({\tau}_{u^{sr}}^{-1}\right);{v}_k^{sr}\sim N\left(0,{\tau}_{v^{sr}}^{-1}\right)\\ {}{\beta}_i^p\sim N\left(0,{\tau}_{\beta^p}^{-1}\right);{\beta}_i^n\sim N\left(0,{\tau}_{\beta^n}^{-1}\right)\\ {}{\tau}_{\ast}^{-1/2}\sim Uniform\left(0,10\right)\end{array}} $$

*α*^*r*^, *α*^*h*^ are the overall mean levels for rodent and human respectively, and assumed to have zero-mean Gaussian prior distributions. The latent variables ${u}_i^h,{u}_i^r,{v}_i^h,{v}_i^r$ are included to model non-temporal background variation with spatial and non-spatial prior distributions. The spatial structure for ${u}_i^h,{u}_i^r$ follow an intrinsic conditional autoregressive (ICAR) [14] model and the non-spatial distribution for ${v}_i^h,{v}_i^r$ is assumed to be zero-mean Gaussian prior distribution. A Gaussian distribution with zero mean is assumed for ${\lambda}_t^h,{\lambda}_t^r$ at *t* = 1 and an autoregressive prior distribution is assumed for ${\lambda}_t^h,{\lambda}_t^r$ at *t* > 1 which allows for a type of nonparametric temporal effect. ${\delta}_{it}^h,{\delta}_{it}^r$ represent the temporal trend for each health district at year *t*. We also assume that the space-time random effects of human and rodent are proportional with one-year lag. This is supported by the finding suggested in [6] that 1-year temporal lag effect can be beneficial to predict human tularemia outbreaks. To model unobserved ecological effects associated with vole cycles at the five boreal zones in Finland (Southern Finland, Southwestern and Inland Finland, Eastern Finland, Northern Finland, and Lapland) [13], two additional parameters ${u}_k^{sr},{v}_k^{sr}$ are included as the spatial and non-spatial contextual variables for regional state level *k.* ${\delta}_{it}^r,{\beta}_i^p,{\beta}_i^n$ are assumed to follow a zero-mean Gaussian prior distribution and the uniform distribution on (0,10) is used to model all standard deviation parameters [15].

Although there is some evidence that a 2-level rodent status could have a lagged predictive ability on human tularemia [6], we also want to extend our consideration to include the original three rodent levels in the joint modeling and assume a categorical likelihood for rodent population status. A specification for multiple categories of rodent status can be defined as

$$ {\displaystyle \begin{array}{l}{\mathrm{h}\mathrm{uman}}_{it}={\mathrm{h}}_{it}\sim Poisson\left({e}_i{\theta}_{it}\right)\\ {}\log \left({\theta}_{it}\right)={\alpha}^h+{u}_i^h+{v}_i^h+{\lambda}_t^h+{\delta}_{it}^h\\ {}{\delta}_{it}^h={\beta}_{i,{r}_{it-1}}{\delta}_{r_{it-1}, it-1}^r\\ {}{\mathrm{r}\mathrm{odent}}_{it}={\mathrm{r}}_{it}\sim Categorical\left({p}_{1, it},{p}_{2, it},{p}_{3, it}\right)\\ {}{p}_{j, it}=\frac{\exp \left({\mu}_{j, it}\right)}{\sum_{j=1}^3\exp \left({\mu}_{j, it}\right)}\\ {}{\mu}_{j, it}={\alpha}^r+{u}_i^r+{v}_i^r+{u}_k^{sr}+{v}_k^{sr}+{\lambda}_t^r+{\delta}_{j, it}^r\end{array}} $$

where *j* = 1 indicates the declining rodent population level, *j* = 2 is the increasing level, and *j* = 3 is the rodent’s population at peak, and ${\delta}_{j, it}^r\sim N\left(0,{\tau}_{\delta^r}^{-1}\right)$. The other prior distributions for the random effects terms are assumed the same as the 2-level model.

We further assume that the space-time random effects of human and rodent are proportional with one-year lag. That is ${\delta}_{it}^h\alpha\;{\delta}_{r_{it-1}, it-1}^r$, i.e. ${\delta}_{it}^h={\beta}_{i,{r}_{it-1}}{\delta}_{r_{it-1}, it-1}^r$ where ${\beta}_{i,{r}_{it-1}}$ is the proportional parameter. This specification is developed to examine the lagged effect of rodent status, ${\delta}_{r_{it-1}, it-1}^r$, at level *j* = *r*_*it*-1_ in health district *i* at time *t-*1on the number of human cases through the space-time interaction term,${\delta}_{it}^h$.

### Model evaluation

To evaluate the models, we use two goodness of fit measures: the Deviance Information Criterion (DIC) [16, 17] where the effective number of parameters is estimated in terms of deviance’s variance, and the Watanabe-Akaike information criterion (WAIC) [18–20]. Both measures are computed under the likelihood of human data to compare the models’ fit with and without the contextual variables. We also compare the posterior standard deviations of *θ*_*it*_ from the two models (binary and categorical) with the rodent data, with those from a model based only human data, to examine the benefits of incorporating animal data in surveillance. Results are obtained from 10,000 posterior samples using WinBUGS software after a burn-in period of 10,000 draws. To assess the mixing of posterior samplers, we adopt Gelman’s $\widehat{R}$ statistics proposed in [18, 21] for multiple chain convergence and converged chains should have the value of $\widehat{R}$ approximately 1.

## Results

**Fig. 3:**
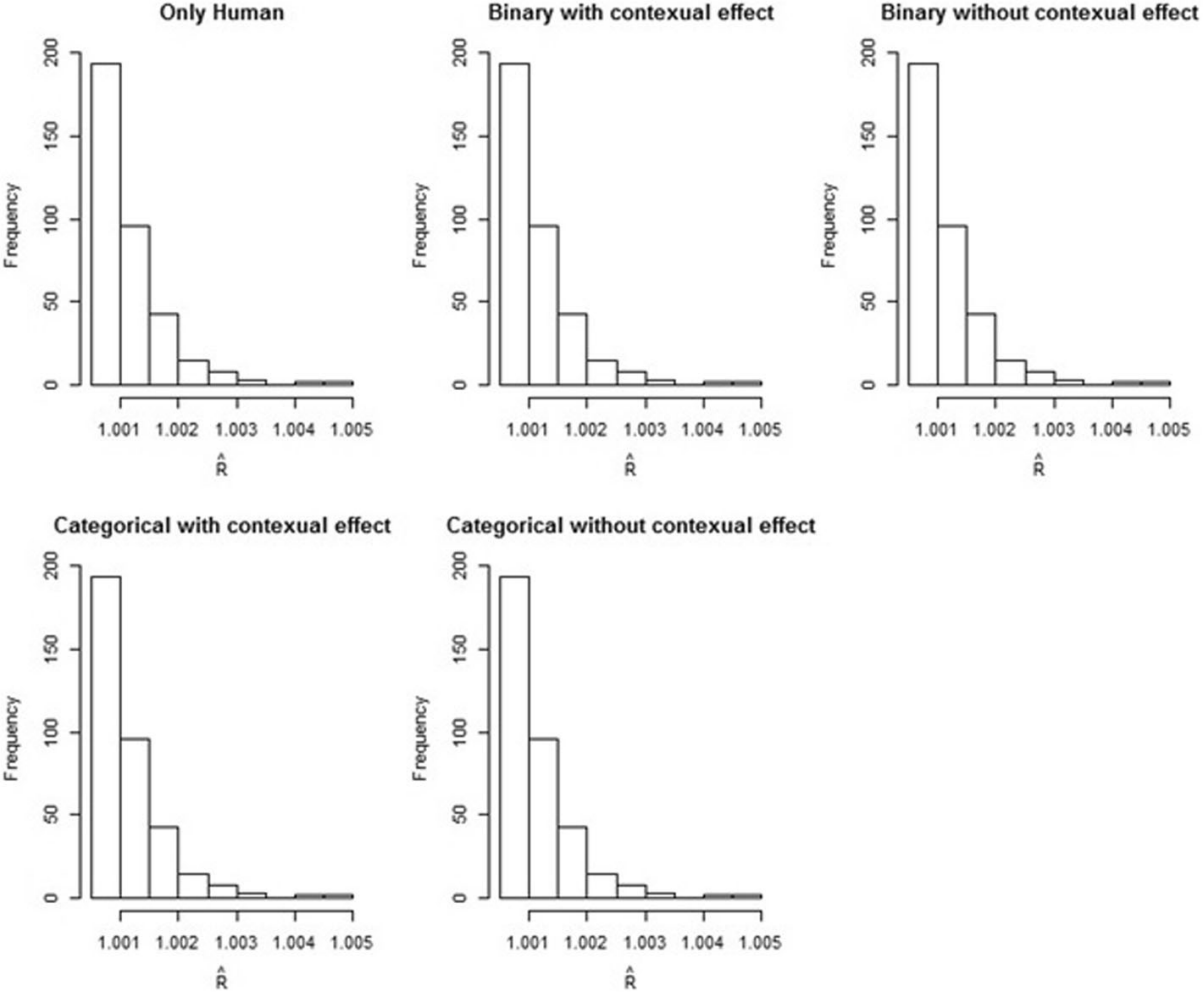
Histograms of $\widehat{R}$ statistics of *θ*_*it*_ from posterior sampler under models

The histograms of $\widehat{R}$ estimates of *θ*_*it*_ from posterior sampler under the models are displayed in Fig. 3. The $\widehat{R}$ estimates under all the models are approximately or less than 1.005 which indicates the chains converge to a posterior distribution. Table 1 displays the DIC and the measures of model performance. We compared the models (binary vs. polytomous) on their DIC and WAIC values under the human likelihood. The binary model without the contextual effect shows the smallest DIC and WAIC values and hence it can be considered as having the best fit to the data. To provide evidence of the benefits from incorporating rodent information into the model, the posterior estimates of standard deviation (SD) of *θ*_*it*_ from both binary and polytomous models are compared and presented in Table 2 for each area over the time period. The SD estimates of *θ*_*it*_ with rodent’s information incorporated are smaller than those from the model that has only human incidence. This result supports the integration of rodent population variables to reduce the uncertainty of tularemia risk estimates.

**Table 1:** DIC (pD) and WAIC (pWAIC) corresponding to the human likelihood for the model comparison

Rodent status	Contextual effect	DIC	pD	WAIC	pWAIC
Binary	With	4269.945	1115.127	3463.587	557.5636
	Without	4262.807	1110.623	3458.687	555.3114
Polytomous	With	4276.408	1123.747	3465.682	561.8733
	Without	4324.426	1170.232	3482.997	585.1162

**Table 2:** Standard deviation of *θ*_*it*_ calculated from posterior samplers for each area over the time period of the models

Health district	Binary		Categorical		Only Human
	With contextual effect	Without contextual effect	With contextual effect	Without contextual effect	
Southwest Finland	1.0216	1.0193	1.1402	1.1569	1.3604
Satakunta	3.6330	3.6124	3.5493	3.5371	3.6365
Kanta-Häme	0.7336	0.7354	0.7632	0.7689	1.0398
Pirkanmaa	3.2779	3.2862	3.2114	3.2071	3.4309
Päijät-Häme	0.9032	0.9011	0.9682	1.0048	1.0475
Kymenlaakso	2.4761	2.4859	2.5092	2.4819	2.7240
South Karelia	0.5381	0.5268	0.5841	0.6060	0.6480
Southern Savonia	0.7490	0.7518	0.7448	0.7722	0.8808
Eastern Savonia	0.7536	0.7551	0.7024	0.6833	0.7279
North Karelia	0.5753	0.5695	0.5475	0.5732	0.5982
Northern Savonia	1.5263	1.5273	1.4859	1.5119	1.6492
Central Finland	5.5499	5.5361	5.5562	5.6234	6.0488
South Bothnia	4.7209	4.7507	4.7506	4.7505	4.7708
Vaasa	2.1997	2.2187	2.2057	2.1912	2.1572
Central Bothnia	3.2251	3.2411	3.2267	3.1927	3.1619
North Bothnia	7.7036	7.6829	7.6786	7.6660	7.5354
Kainuu	0.1984	0.1957	0.2255	0.2468	0.2596
Länsi-Pohja	0.7437	0.7415	0.7377	0.7399	0.6897
Lapland	0.4929	0.4905	0.4979	0.5332	0.6323
Helsinki and Uusimaa	3.3256	3.3243	3.3982	3.4146	3.8419
Average over all areas	2.2174	2.2176	2.2242	2.2331	2.3421

**Fig. 4:**
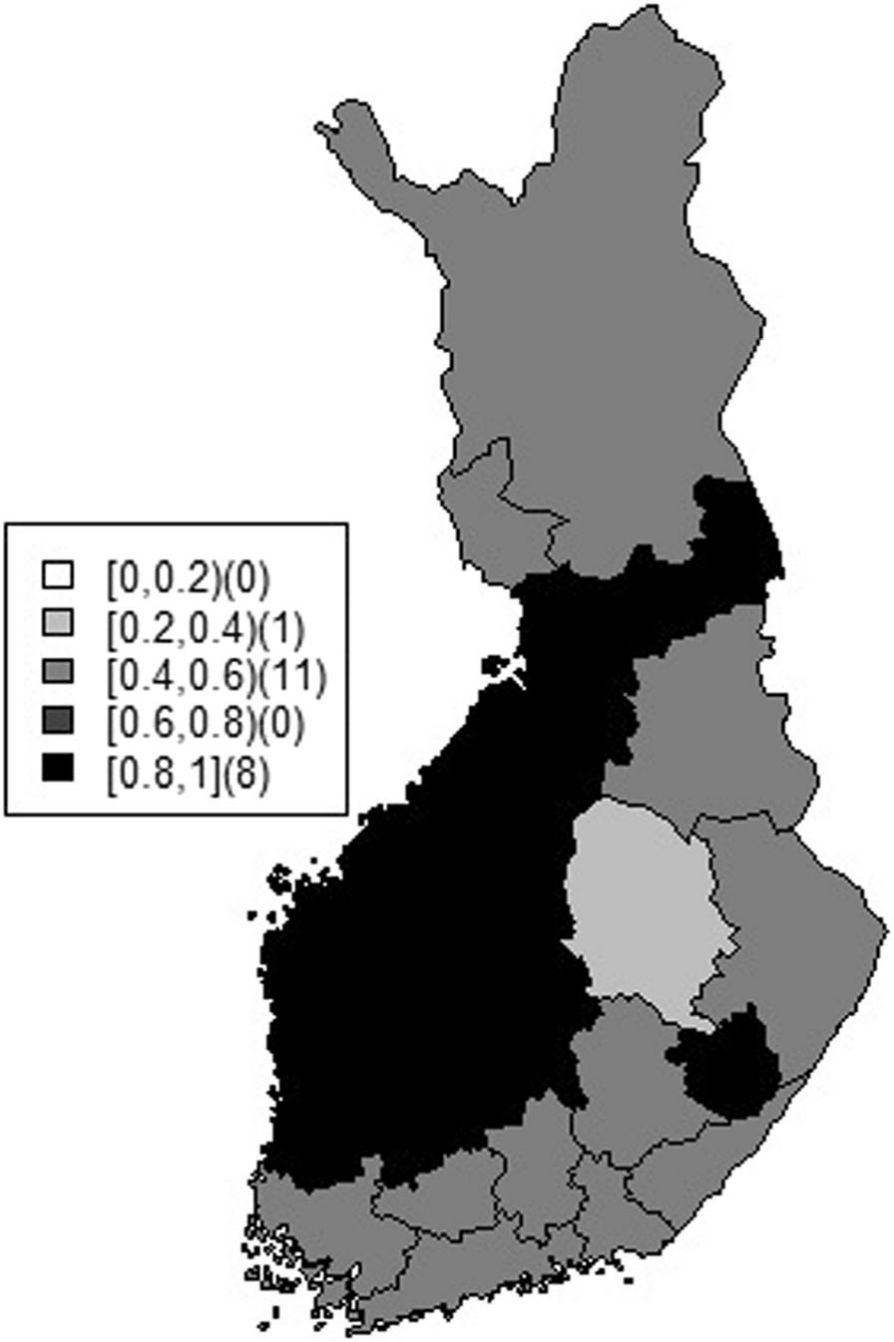
Map of mean of DP under the binary rodent model without the contextual effect for each health district

To help assess the predictive performance of rodent abundance data on the occurrence of human cases, we develop a metric on the 0 to 1 scale, namely the degree of positive indicator (DP) for each health district as ${DP}_i=\frac{\exp \left({\beta}_i^p\right)}{\exp \left({\beta}_i^p\right)+1}.$ The DP indicator is derived from the binary model without the contextual factors as this was the model with the lowest DIC and WAIC. High values of DP (close to 1) would indicate health districts with a high number of human tularemia cases in the current year, given increasing or at peak rodent populations in the past year. DP can be interpreted in a similar fashion to the sensitivity of a diagnostic test. High values of DP would suggest good predictive value of the rodent data on the occurrence of human tularemia cases. Year-specific modeling was not considered due to insufficient data. Thus positive dependence values represent an averaged effect of rodent populations on human incidence across all the years (Table 3). There are 8 (40%) health districts, mostly on the west and south of the country, with mean DP values larger than 0.8 and the lower 95% credible interval above 0.5 (Fig. 4).

**Table 3:** The mean values and 95% credible intervals (CrI) of DP under the binary rodent model without the contextual effect for 20 health districts

Health district	Lower 95% CrI	Mean	Upper 95% CrI
Southwest Finland	0.0129	0.4974	0.9881
Satakunta	0.8447	0.9686	1.0000
Kanta-Häme	0.0019	0.5099	0.9985
Pirkanmaa	0.7794	0.9428	0.9996
Päijät-Häme	0.0037	0.4921	0.9951
Kymenlaakso	0.0000	0.5057	1.0000
South Karelia	0.0004	0.5031	0.9996
Southern Savonia	0.0001	0.5010	1.0000
Eastern Savonia	0.9407	0.9929	1.0000
North Karelia	0.0000	0.5005	1.0000
Northern Savonia	0.0006	0.3543	0.9965
Central Finland	0.9485	0.9931	1.0000
South Bothnia	0.9276	0.9901	1.0000
Vaasa	0.9621	0.9953	1.0000
Central Bothnia	0.9755	0.9973	1.0000
North Bothnia	0.8998	0.9832	1.0000
Kainuu	0.0004	0.5060	0.9992
Länsi-Pohja	0.0000	0.5789	1.0000
Lapland	0.0013	0.5064	0.9993
Helsinki and Uusimaa	0.0347	0.4788	0.9568

## Discussion

Other modifications are possible. For example, in joint modeling human and rodent data we could consider human cases being dependent on rodent population through *p*_*it*_ as a categorical covariate. For spatial unit, smaller or administrative areas different from health districts may have led to more discriminatory findings (or not given increased noise from smaller units). Similarly, more informative data on rodent population levels, e.g. rodent densities as in other studies [11] might have resulted in more descriptive models. Rodent populations fluctuate with a highly varying amplitude, which means that abundances may vary substantially from one peak to the next, even within the same regions [12]. If tularemia transmission to humans is a phase and density dependent process, as it most likely is [6], variation in vole abundance during successive peaks may reduce the predictive value of models employed here. Subsequent studies must explore the incorporation of data on the precise location of the rodent trapping sites through the use of some form of interpolation [22]. In addition to host population changes, environmental factors also seem to impact on the occurrence of tularemia outbreaks [23, 24]. Incorporation of evidence on mosquito distribution (not purposely captured at this moment in Finland), rainfall and water bodies into our models would be straightforward.

## Conclusions

Space-time proximity and contact patterns between humans and animals play a central role in infection risk. In this research, we attempted to assess the surveillance relevance of regularly collected rodent population data to i) improve tularemia risk estimates and ii) inform early prediction of human tularemia cases in Finland. To that effect, we developed binary and polytomous architectures to jointly model human incidence and rodent status with one-year lag. Our results returned a heterogeneous picture but for many health districts rodent population status was relevant to the occurrence of human tularemia cases. We have shown that the incorporation of rodent population data led to an improvement in the accuracy of human risk estimates in 15 (75%) health districts, compared to models only considering human tularemia cases. Furthermore, our purposely built indicator (DP) showed that in 8 (40%) of the health districts, increasing and at-peak rodent populations were robust predictors of human tularemia cases in the following year. However, for few districts (e.g. Länsi-Pohja and Kainuu) where the model based only on human data leads to more precise estimates of incidence, these areas also have the low values of positive indicator (DP) with the corresponding credible intervals crossing 0.5. This suggests that the distribution of zoonotic pathogens in animal and human populations spatially varies as we assumed according to local biotic and abiotic determinants. To conduct further investigation, we need more information on several covariates such as environmental, behavioral, and socio-economic factors. However, the platform proposed in this research can facilitate in identification of geographical areas that are potentially suitable for transmission.

Our objective was to develop different models to combine multiple data sources already available, on animals and humans, to better inform the occurrence of zoonoses. The present work shows the utilization of animal population data (in the absence of animal health-related data on rodents) to inform human risk. Although different model parameterizations and, in particular, evidence on other putative predictors, as reported elsewhere [25], could contribute further evidence to better inform human risk, our proposed methodology demonstrates its ability to quantify the association between the rodent status and human incidence. This is potentially useful in prediction of human outbreak when put in the health-policy perspective.

## Abbreviations

CrI: Credible interval
DIC: Deviance Information Criterion
DP: Degree of positive indicator
ICAR: Intrinsic conditional autoregressive model
SD: Standard deviation
WAIC: Watanabe-Akaike information criterion

## References

[1] Vrbova L (2016). Utility of algorithms for the analysis of integrated Salmonel la surveillance data. Epidemiol Infect.

[2] Wendt A, Kreienbrock L, Campe A. Joint use of disparate data for the surveillance of Zoonoses: a feasibility study for a one health approach in Germany. Zoonoses Public Health. 2016;63(7):503–14. 10.1111/zph.12255 26812912

[3] Rossow H (2014). Risk factors for pneumonic and ulceroglandular tularaemia in Finland: a population-based case-control study. Epidemiol Infect.

[4] Eliasson H (2002). The 2000 tularemia outbreak: a case-control study of risk factors in disease-endemic and emergent areas, Sweden. Emerg Infect Dis.

[5] Dennis DT (2001). Tularemia as a biological weapon: medical and public health management. Jama.

[6] Rossow H, et al. Incidence and seroprevalence of tularaemia in Finland, 1995 to 2013: regional epidemics with cyclic pattern. Euro Surveill. 2015;20(33). 10.2807/1560-7917.ES2015.20.33.21209 10.2807/1560-7917.es2015.20.33.21209 26314404

[7] Tärnvik A, Sandström G, Sjöstedt A (1996). Epidemiological analysis of tularemia in Sweden 1931–1993. FEMS Immunol Med Microbiol.

[8] Reintjes R (2002). Tularemia outbreak investigation in Kosovo: case control and environmental studies. Emerg Infect Dis.

[9] Allue M, et al. Tularaemia outbreak in Castilla y León, Spain, 2007: an update. Euro Surveill. 2008;13(32) 18761900

[10] Grunow R (2012). Surveillance of tularaemia in Kosovo*, 2001 to 2010.

[11] Luque-Larena JJ (2015). Tularemia outbreaks and common vole (Microtus arvalis) irruptive population dynamics in northwestern Spain, 1997–2014. Vector Borne Zoonotic Dis.

[12] Korpela K (2013). Nonlinear effects of climate on boreal rodent dynamics: mild winters do not negate high-amplitude cycles. Glob Chang Biol.

[13] Sane J, et al. Regional differences in long-term cycles and seasonality of Puumala virus infections, Finland, 1995–2014. Epidemiol Infect. 2016;144(13):2883–2888. 10.1017/S0950268816000765 PMC9150413 27113030

[14] Besag J. Spatial interaction and the statistical analysis of lattice systems. J R Stat Soc Ser B Methodol. 1974:192–236.

[15] Gelman A (2006). Prior distributions for variance parameters in hierarchical models (comment on article by Browne and Draper). Bayesian Anal.

[16] Spiegelhalter DJ (2002). Bayesian measures of model complexity and fit. J R Stat Soc Series B Stat Methodol.

[17] Celeux G (2006). Deviance information criteria for missing data models. Bayesian Anal.

[18] Gelman A (2014). Bayesian data analysis.

[19] Gelman A, Hwang J, Vehtari A (2014). Understanding predictive information criteria for Bayesian models. Stat Comput.

[20] Watanabe S (2013). A widely applicable Bayesian information criterion. J Mach Learn Res.

[21] Brooks SP, Gelman A (1998). General methods for monitoring convergence of iterative simulations. J Comput Graph Stat.

[22] Diggle PJ, Menezes R, Su Tl (2010). Geostatistical inference under preferential sampling. J R Stat Soc: Ser C: Appl Stat.

[23] Faith S (2012). Growth conditions and environmental factors impact aerosolization but not virulence of Francisella tularensis infection in mice. Front Cell Infect Microbiol.

[24] Leblebicioglu H (2008). Outbreak of tularemia: a case–control study and environmental investigation in Turkey. Int J Infect Dis.

[25] Ariza-Miguel J (2014). Molecular investigation of tularemia outbreaks, Spain, 1997–2008. Emerg Infect Dis.

